# Supplementary material for: Calcareous Materials Effectively Reduce the Accumulation of Cd in Potatoes in Acidic Cadmium-Contaminated Farmland Soils in Mining Areas
Source: Int J Environ Res Public Health. 2022 Sep 17;19(18):11736. doi: 10.3390/ijerph191811736 (PMC9517293; doi:10.3390/ijerph191811736)
Supplement: Supplementary file 1 [file ijerph-19-11736-s001.zip › ijerph-1901020-supplementary.pdf]

Supplementary Material

Supplementary Table S1. The effect of different passivation materials on the physical and chemical properties of soil (n=144).

heavy metal elements	Max. (mg/kg)	Min. (mg/kg)	Avg. (mg/kg)	Background value. (mg/kg)	Risk screening value. (mg/kg)
Cr	122.1	45.7	87.2	95.9	150
Ni	98.4	33.8	60.9	39.1	70
Cu	66.1	25.7	45.2	32.0	50
Zn	182.5	85.1	139.9	99.5	200
Cd	3.1	1.1	1.9	0.659	0.3
Pb	48.2	21.2	34.5	35.2	90

The risk screening value refers to the Chinese soil environmental quality standard (GB15618-2018).

Supplementary Table S2. The effect of different passivation materials on the physical and chemical properties of soil (n=42).

Physical and Chemical Index of Soil	Max.	Min.	Avg.	Standard deviation	Coefficient of Variation
Organic matter/（g/kg）	26.55	31.67	27.73	1.26	0.05
Total N/ （g/kg）	1.69	1.83	1.76	0.04	0.02
Alkaline N/ （g/kg）	1.00	1.29	1.13	0.08	0.07
Total P/（g/kg）	1.65	2.22	2.04	0.15	0.08
Available P/（g/kg）	0.25	0.33	0.29	0.02	0.07
Total K/（g/kg）	11.8	13.6	13.09	0.41	0.03
Available k/（g/kg）	1.93	2.97	2.28	0.33	0.15
Clay/（%）	39.4	49.2	45.06	2.62	0.06
Slit/（%）	21.7	26.53	23.23	1.22	0.05
Sand/（%）	27.73	36.93	31.71	2.68	0.08
Total Si/（g/kg）	61.5	75.2	69.96	3.98	0.06
Total Mg/（g/kg）	5.00	5.90	5.50	0.22	0.04
Total Fe/（g/kg）	51.5	60	58.04	1.94	0.03
Total Mn/（g/kg）	0.67	0.81	0.77	0.04	0.05
Total Al/（g/kg）	9.50	12.90	11.54	1.03	0.09
